# Log file‐based treatment efficiency analysis of TrueBeam and Halcyon delivery systems

**DOI:** 10.1002/acm2.70802

**Published:** 2026-09-14

**Authors:** Ruiming C. Edmondson, Douglas J. Moseley, Satomi Shiraishi, James A. Kavanaugh

**Affiliations:** ^1^ Department of Radiation Oncology Mayo Clinic Rochester Minnesota USA

**Keywords:** Treatment efficiency, Trajectory logs

## Abstract

**Background:**

TrueBeam (TB) and Halcyon (HC) are state‐of‐the‐art linear accelerators (linacs) widely utilized for external beam radiotherapy. As clinical demand increases, expanding treatment capacity through new machine installations or extended operating hours remains costly and resource intensive. Retrospective analyses of treatment delivery performance can help guide machine assignment strategies that optimize machine utilization, reduce patient time alone on the treatment couch, and improve overall departmental throughput.

**Purpose:**

To introduce a trajectory log‐based methodology for assessing treatment delivery efficiency on Halcyon and TrueBeam systems by quantifying gantry speed, MLC speed, and dose‐rate utilization, and to integrate these machine‐level parameters with beam‐on time and modulation factor (MF) to guide treatment machine assignment and patient triaging.

**Methods:**

Volumetric modulated arc therapy (VMAT) trajectory logs acquired between February 2025 and February 2026 were collected from fourteen clinical linacs across a main institution and its affiliated regional clinics. The percentages of beam‐on time during which the machine operated at ≥95% of its maximum dose rate (max_DR), gantry speed (max_GS), or MLC speed (max_MLC) were quantified. Plans were categorized by procedure type and disease site, and delivery efficiencies were compared using Mann‐Whitney U test.

**Results:**

A total of 4347 VMAT plans were included in the study. For stereotactic body radiation therapy (SBRT) plans, Halcyon had a significantly higher max_DR (0.96 ± 0.14 vs. 0.51 ± 0.39 for TrueBeam), suggesting its max dose rate might be a bottleneck for treatment efficiency. For non‐SBRT plans, Halcyon demonstrated greater max_GS (0.68 ± 0.32 vs. 0.43 ± 0.22 for TrueBeam) and max_MLC (0.66 ± 0.32 vs. 0.38 ± 0.23 for TrueBeam), which is reflected in its higher MF and faster beam‐on time compared to TrueBeam, saving over 50 seconds of on‐table time per plan for each patient.

**Conclusions:**

Halcyon demonstrates superior delivery efficiency for non‐SBRT plans compared to TrueBeam through higher usage of its maximum MLC speed and gantry speed throughout treatment, especially for pelvis plans. In contrast, TrueBeam has significantly shorter beam‐on time for higher‐dose treatments (e.g. prostate SBRT), likely due to its higher nominal maximum dose rates at flattening filter‐free energies. By linking delivery performance to underlying machine‐specific limitations, this work provides a transferable framework for machine efficiency assessment that can be readily implemented at other centers using trajectory log data to guide machine assignment decisions and evaluate the potential impact of future Linac technology advancements.

## 1 INTRODUCTION

Modern radiation oncology clinics face increasing treatment demands as global cancer incidence continues to rise, with approximately 20 million new cases diagnosed annually and projections approaching 35 million cases by 2050.^1^ This growth places increasing challenges on radiation oncology departments to improve treatment capacity and clinical throughput;^2^ however, for high‐volume clinics, adding new incremental linear accelerators (linacs) or extending operating hours could be costly and resource‐intensive. Alternatively, clinics operating multiple types of external beam delivery systems may benefit from optimized machine assignment workflows that maximize treatment efficiency based on patient‐specific treatment sites and techniques. Retrospective analysis of treatment delivery statistics provides a data‐driven approach to designing machine assignment and patient triaging strategies that lower on‐table time to improve clinical throughput, mitigate patient stress, and leverage existing clinical resources during normal operating hours by maximizing the capabilities of available treatment delivery systems.

TrueBeam (TB) and Halcyon (HC) (Varian Medical Systems, Palo Alto, CA) are two types of the state‐of‐the‐art linacs that are currently implemented in clinics as photon external beam radiotherapy delivery systems. The TB is a versatile C‐arm Linac offering multiple photon and electron energies, high dose rate flattening filter free (FFF) beams, and hardware configurations suitable for a broad range of treatment techniques.^3^ The Edge system shares the same platform but features high‐resolution 2.5‐mm multileaf collimator (MLC) leaves, making them ideal to deliver high precision treatments such as stereotactic body radiation therapy (SBRT) and stereotactic radiosurgery (SRS) procedures.^4^

Halcyon, by contrast, employs an O‐ring gantry design with a dual‐layer, stacked and staggered MLC and fixed 6 MV FFF beam energy. While it provides substantially higher maximum gantry rotation speed and MLC leaf speeds compared with TB, its single energy and single dose rate design limits flexibility for certain clinical scenarios.^5^ Prior feasibility studies have demonstrated that HC can achieve non‐inferior plan quality for volumetric modulated arc therapy (VMAT) across multiple disease sites, including breast^6^ and lung SBRT plans^7^ compared to TBs. Other investigations have reported lower doses to organs at risk (OARs) and improved gamma passing rates on HC, albeit sometimes with longer overall treatment times.^8^, ^9^ Additional work has shown Halyon to have similar MLC positional accuracy and superior gantry angle accuracy relative to TB,^10^ along with early evidence of comparable clinical outcomes.^11^

Linac trajectory logs are automatically recorded snapshots of machine parameters during treatment delivery.^12^ These logs contain static plan information as well as dynamic axis data captured at 20‐ms intervals, including gantry angle, collimator position, couch coordinates, monitor unit (MU) delivered, and individual MLC leaf positions. Previous studies have demonstrated that trajectory logs can accurately characterize MLC positioning error^13^ and assess dosimetric accuracy for VMAT deliveries.^14^ Beyond treatment delivery analysis, trajectory logs have also been utilized for patient‐specific QA, routine MLC QA, and assessment of machine performance trends.

Treatment delivery efficiency has been previously assessed using treatment planning system (TPS) generated or static machine delivery metrics including beam‐on/treatment time, number of treatments per day, planning modulation factor (MF), and total MU.^15^ However, there remains a lack of a comprehensive multi‐metric assessment of dynamic machine‐specific parameters (e.g. instantaneous MLC speed, gantry speed, and dose rate) throughout treatment that fundamentally drive delivery efficiency. These parameters are continuously adjusted by the Linac control system through real‐time trade‐offs during treatment and are not explicitly encoded in the TPS‐generated DICOM RT plan, necessitating analysis of actual machine delivery data. In this single‐institution multi‐site study, we introduce a trajectory log‐based methodology to quantify the utilization of these machine parameters during VMAT delivery to better understand the underlying drivers of treatment efficiency. These treatment efficiency metrics are then assessed across multiple disease sites and treatment types (SBRT/SRS vs. non‐SBRT) for TB and HC systems in a large clinical cohort treated over a one‐year period, expanding upon previous studies through both scale and comprehensive subgroup analyses. These results aim to provide an institutionally transferrable methodology which uses readily available trajectory logs to identify machine‐specific performance trends that may inform patient assignment strategies and support optimized clinical treatment throughput.

## 2 METHODS

VMAT trajectory logs from February 2025 to February 2026 were collected from fourteen linacs (two HCs, 11 TBs, and one Edge) across a main institution and its affiliated regional clinics. All treatment plans were generated in the Eclipse™ v18.0.1 TPS and had passed IMRT QA prior to clinical delivery. One delivered fraction from each unique treatment plan was randomly selected and included for analysis. Dose per fraction and treatment type (SBRT/SRS vs. non‐SBRT) associated with each log were retrospectively extracted from the clinical record‐and‐verify system.

Trajectory log information for each plan (plan name, machine, energy, beam‐on time, number of arcs, and MU) was collected using an in‐house MATLAB tool (R2024b, MathWorks, Natick, MA). MFs were calculated as the MU divided by prescribed dose (in cGy) per fraction. Dynamic axis data (at 20‐ms interval) of MU delivered, gantry angle, and individual MLC leaf positions, were used to compute instantaneous dose rate, gantry speed, and MLC speed using a central finite difference methodology.^16^ A 3‐sample boxcar filter was used before numerical differentiation to minimize the impact of digitization noise. For plans delivered without auto‐sequencing of sub‐beams, trajectory logs from all sub‐beams associated with the plan were concatenated to capture the complete delivery sequence.

To quantify machine efficiency during treatment delivery, the fraction of beam‐on time where the dose rate, gantry speed, or MLC speed reaches ≥ 95% of the machine specified capabilities was calculated. They are referred to as delivery efficiency metrics of max_DR, max_GS, and max_MLC, respectively, and have values ranging from 0 to 1 each. To assess robustness of this metric across different treatment days/fractions and different machines, trajectory log information and treatment efficiency calculations were compared for a subset of randomly selected plans across all fractions, including fractions where the plan was treated on different (dosimetrically matched) machines. Additionally, to assess data analysis fidelity, a subset of trajectory logs were read out independently using Log Analyzer of pylinac^17^ 3.45.0, and the associated delivery efficiency metrics were calculated and validated separately using Python (version 3.12; Python Software Foundation, Wilmington, DE).Lastly, trajectory logs of a prostate SBRT plan that has been planned and treated on both HC and TB were selected for a direct comparison of treatment efficiency under identical patient anatomy.

For statistical analysis, each plan was subcategorized according to their machine type (TB vs. HC), procedure type (SBRT/ SRS vs. non‐SBRT), and disease site (pelvis, abdomen, prostate, head and neck). The TB and Edge plans were grouped together due to their nearly identical hardware configurations and manufacturer specifications aside from MLC width and field size (MLC leaf travel speed limits, however, remain the same for both systems). Mann‐Whitney U test was used to assess statistical significance for all comparisons, with significance defined as *p* < 0.05.

## 3 RESULTS

**FIGURE 1:**
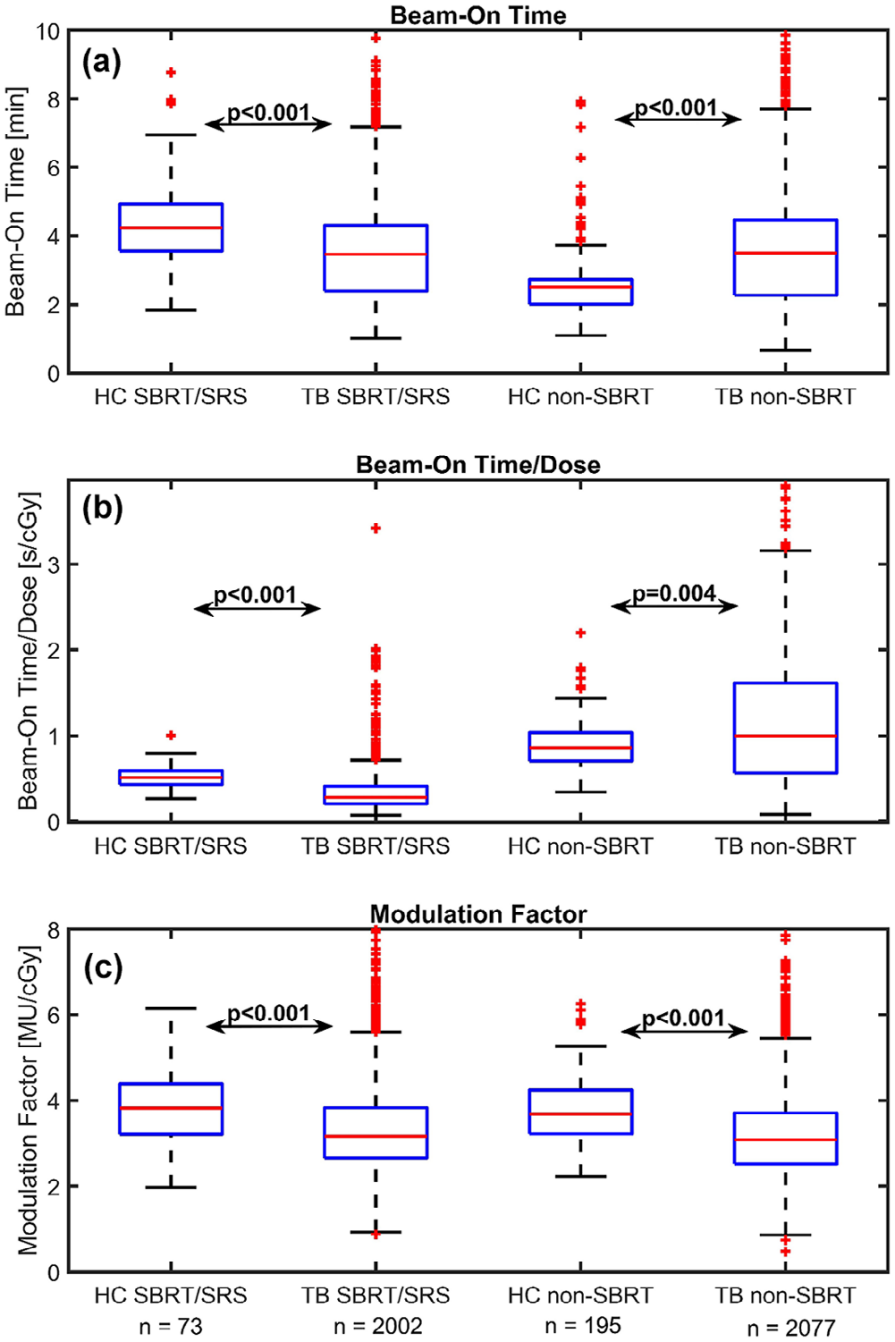
(a) Beam‐on time, (b) beam‐on time normalized by dose per fraction, and (c) MF for HC and TB, grouped by treatment category (SBRT/SRS vs. non‐SBRT).

A total of 4347 unique VMAT plans of 3286 patients were included in the analysis. Table 1 summarizes the distribution of trajectory logs across the defined subcategories. SBRT/SRS plans had median of 1000 cGy per fraction, whereas non‐SBRT plans had median of 300 cGy per fraction. Trajectory log file structure and delivery information remained consistent across all treatment days and fractions for a given plan, including instances in which the plan was delivered on a different dosimetrically matched machine. Overall, in comparison to TB, delivered HC plans demonstrated significantly higher MFs (3.79 ± 0.79 HC vs. 3.29 ± 1.07 TB) and higher number of arcs (3.42 ± 0.55 HC vs. 3.01 ± 0.64 TB). For SRS/SBRT plans, HC exhibited significantly longer beam‐on times (> 40s) than TB (4.38 ± 1.25 min HC vs. 3.58 ± 1.49 min TB), and this relationship remained consistent when beam‐on time was normalized by prescribed dose per fraction. In contrast, for non‐SBRT plans, HC demonstrated > 50 s reduction in beam‐on time compared to TB (2.67 ± 1.13 min HC vs. 3.53 ± 1.70 min TB), with the same trend observed after dose normalization (Figure 1a and b). Additionally, HC plans had significantly higher MF than TB for both procedure categories (Figure 1c).

**TABLE 1:** Trajectory log subcategories and the corresponding number of plans included in the analysis.

Characteristic	TB (*n* = 4079)	HC (*n* = 268)
Energy		
6 MV	1501	–
6 MV FFF	1264	268
10 MV FFF	1314	–
Procedure		
SBRT/SRS	2002	73
Non‐SBRT	2077	195
Treatment Site		
Pelvis	741	133
Abdomen	242	27
Prostate	224	74
Head & Neck	156	32

**FIGURE 2:**
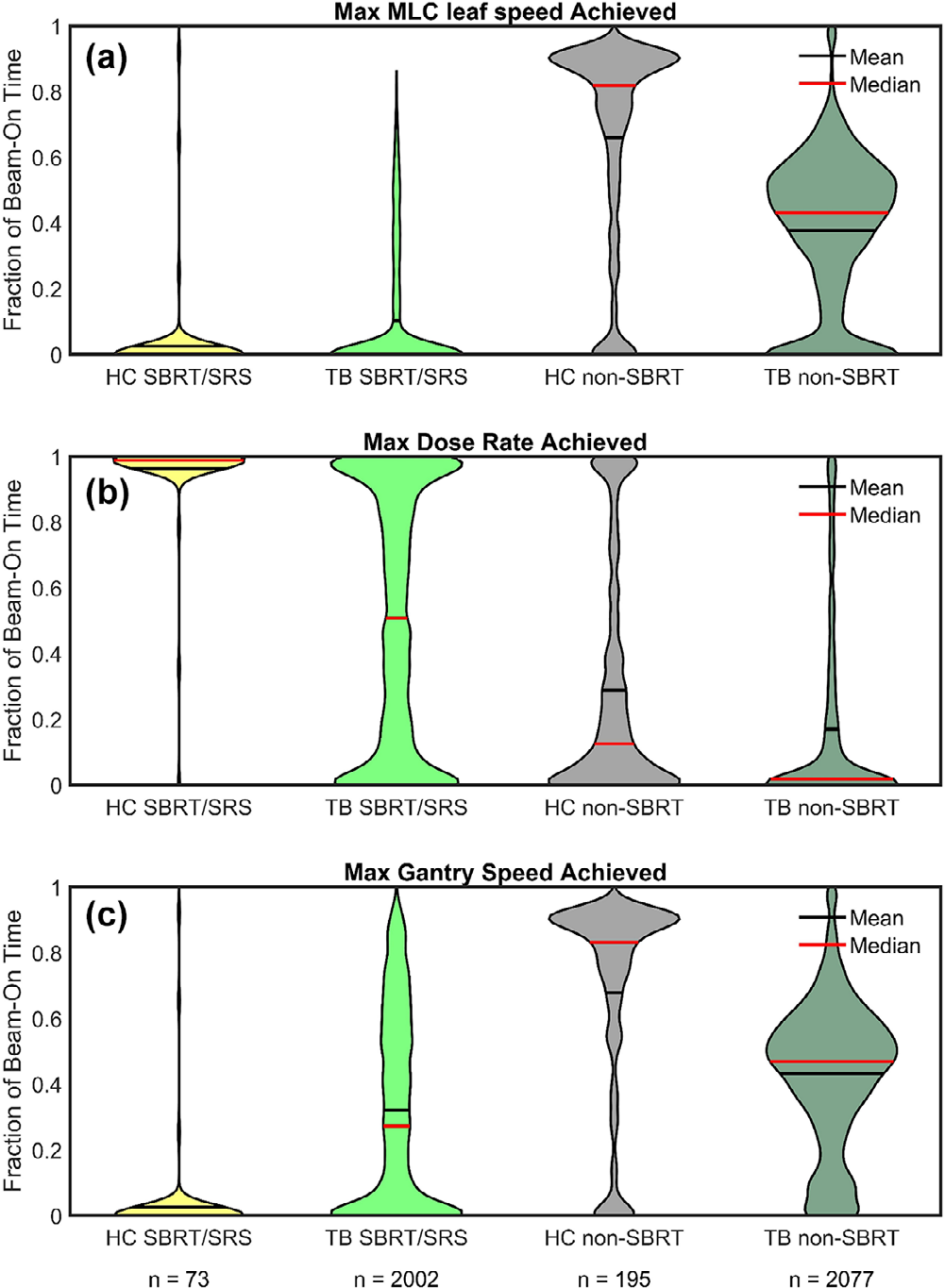
Fraction of beam‐on time during which ≥95% of the maximum (a) MLC speed, (b) dose rate, and (c) gantry speed was achieved for HC and TB, grouped by treatment type (SBRT/SRS vs. non‐SBRT).

For both delivery systems, SBRT/SRS plans achieved significantly greater max_DR values, whereas non‐SBRT plans demonstrated significantly greater max_GS and max_MLC values (Figure 2). For SBRT/SRS plans, HC had near maximal max_DR utilization (0.96 ± 0.14 HC) compared with TB (0.51 ± 0.39 TB), suggesting that maximum dose rate may represent a limiting factor for treatment efficiency on the HC platform. In contrast, max_GS and max_MLC values were significantly lower for both delivery systems, suggesting potentially reduced utilization of gantry and MLC modulation capabilities to prioritize maximal dosimetric throughput. For non‐SBRT plans, HC demonstrated significantly greater max_GS (0.68 ± 0.32 HC vs. 0.43 ± 0.22 TB) and max_MLC (0.66 ± 0.32 HC vs. 0.38 ± 0.23 TB) compared with TB, reflecting a greater tendency for the HC system to maximize delivery efficiency through gantry and MLC motion. Overall, plans with high max_DR values tended to exhibit lower utilization of gantry and MLC speeds, and vice versa, suggesting a trade‐off between dose rate optimization and delivery modulation. Table 2 provides a comprehensive comparison of the three delivery efficiency metrics between the HC and TB systems.

**TABLE 2:** SBRT versus. non‐SBRT delivery efficiency metrics for the two treatment systems. Max_GS, max_MLC, and max_DR correspond to the percentages of beam‐on time during which the machine operated at ≥95% of its gantry speed, multileaf collimator (MLC) leaf speed, or dose rate, respectively.

Treatment Type	Max_GS	Max_MLC	Max_DR
SBRT			
HC	0.03 ± 0.13	0.03 ± 0.13	0.96 ± 0.14
TB	0.32 ± 0.29^*^	0.10 ± 0.19^*^	0.51 ± 0.39^*^
Non‐SBRT			
HC	0.68 ± 0.32	0.66 ± 0.32	0.29 ± 0.35
TB	0.43 ± 0.22^*^	0.38 ± 0.23^*^	0.17 ± 0.28^*^

* Statistical significance (*p* < 0.001) between the HC and TB for each subcategory of SBRT and non‐SBRT treatments.

**FIGURE 3:**
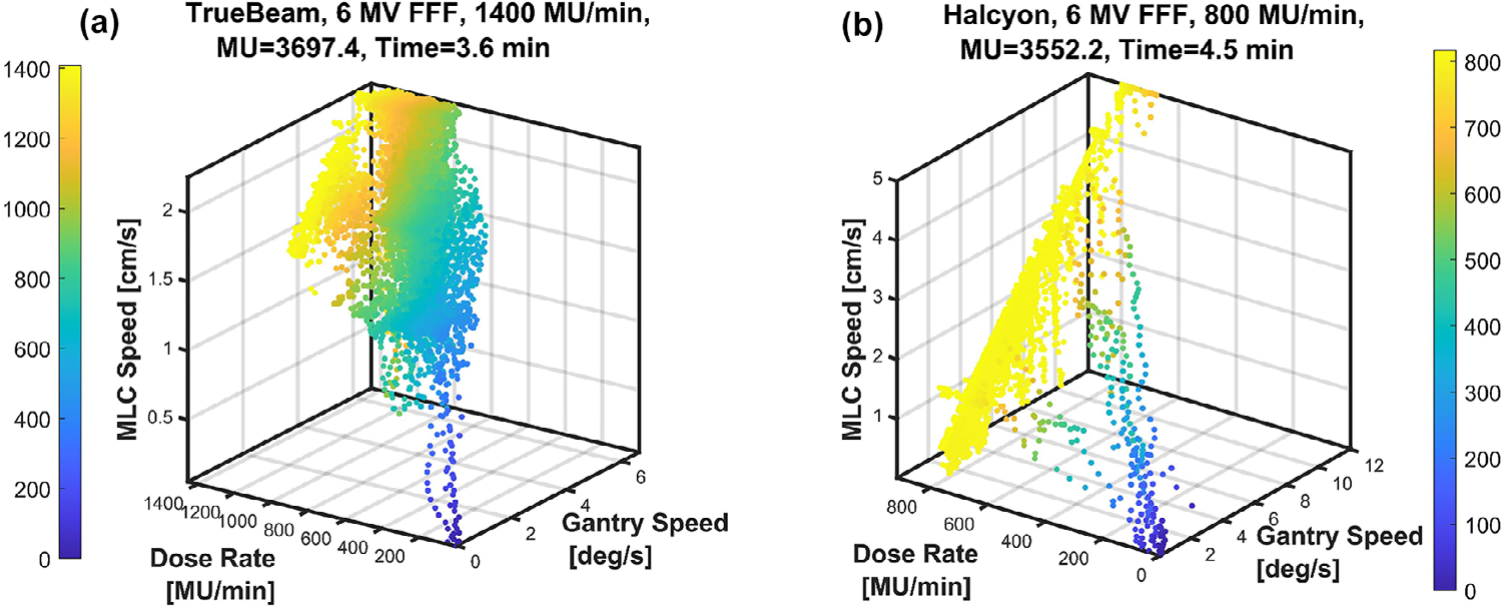
Instantaneous gantry speed, dose rate, and MLC leaf speed estimated at 20‐ms intervals (each represented by a scatter point) for (a) TrueBeam and (b) The HC prostate SBRT trajectory logs used for the same patient's treatment delivery. Color scale ranges from 0 to the sum of the maximum values of gantry speed, dose rate, and MLC leaf speed.

Figure 3 provides a representative clinical example illustrating these system‐specific delivery characteristics. A prostate SBRT case was initially planned and delivered on HC for the first fraction and subsequently replanned and delivered on TB for the remaining four fractions to utilize its 6DoF couch for improved patient alignment. The same 6 MV FFF energy (max dose rate = 800 MU/min and 1400 MU/min for HC and TB, respectively) was used for both plans. The 3D visualizations of instantaneous MLC speed, gantry speed, and dose rate demonstrate that the HC plan operated at its maximum dose rate for a significantly larger fraction of the delivery (max_DR = 0.98 and 0.12 for HC and TB, respectively), whereas the TB plan exhibited significantly greater utilization of gantry and MLC speeds throughout treatment (max_GS = 0.34 TB and max_MLC = 0.39 TB) compared with HC (max_GS = 0.005 HC vs. max_MLC = 0.007 HC). Interestingly, despite the absence of apparent dose rate limitation, TB did not achieve maximal utilization of MLC and gantry speed in this case. This example highlights how treatment efficiency can be governed by different machine constraints. These differences resulted in a shorter beam‐on time for the TB plan, but with a lower MF compared to HC.

**FIGURE 4:**
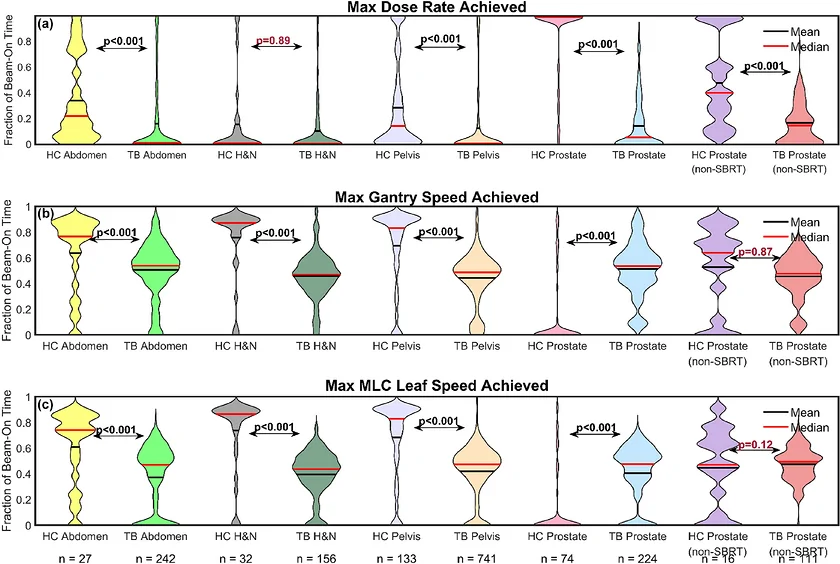
Fraction of beam‐on time during which ≥95% of the maximum (a) dose rate, (b) gantry speed, and (c) MLC leaf speed was achieved for HC and TB, grouped by disease sites of abdomen, head and neck (H&N), pelvis, prostate (all plans), and prostate (non‐SBRT only).

Machine delivery efficiency parameters grouped by disease sites (Figure 4) demonstrate significantly greater utilization of gantry and MLC speeds by Halcon in head and neck, abdomen, and pelvis. In contrast, for prostate cases, HC exhibits significantly higher max_DR but significantly lower max_GS and max_MLC compared to TB. After excluding prostate SBRT plans, differences in max_GS and max_MLC were no longer significant (*p* = 0.87 and 0.12, respectively). Interestingly, HC demonstrated significantly higher utilization of all three machine parameters than TB for abdomen and pelvis cases. HC had significantly shorter beam‐on times than TB for all disease sites except prostate, where TB demonstrated shorter beam‐on times, as shown in Figure S1. After excluding prostate SBRT cases, no statistically significant difference in beam‐on time was observed, consistent with the comparable gantry and MLC speed utilization between systems.

## 4 DISCUSSION

This study introduces a transferrable methodology which uses readily available trajectory logs to provide assessment and comparison of treatment delivery efficiency between HC and TB systems. Additionally, to build upon prior studies that investigated TPS‐generated delivery metrics of smaller cohorts and selected disease sites, this work analyzes a substantially larger dataset of over 4000 clinically delivered plans and directly quantifies the machine‐level parameters that drive delivery efficiency, including instantaneous dose rate, MLC speed, and gantry speed throughout treatment delivery. In addition, comprehensive subgroup analyses were performed across multiple disease sites and treatment techniques for the two external beam delivery platforms, providing a detailed characterization of how the two systems operate in routine clinical practice.

Analysis of instantaneous gantry speed, MLC speed, and dose rate revealed a clear trade‐off among the three machine parameters. High‐dose‐rate operation (e.g. SBRT) requires reduced gantry and MLC speeds to support sufficient MU deposition, whereas highly modulated, larger target, lower‐dose‐rate plans (e.g., head and neck) demand rapid gantry and MLC motion, resulting in limited use of maximum dose rate. Neither delivery platform operated near its maximum for all three parameters concurrently. The trade‐offs between these three delivery parameters are determined by the Linac control system and not explicitly embedded in the TPS‐generated DICOM RT file, requiring the evaluation of post‐delivery log‐files to determine the speed across all the parameters.

For SBRT/SRS plans, the instantaneous dose rate analysis shows HC's lower nominal maximum dose rate (800 MU/min vs. 1400 MU/min for 6 MV FFF and 2400 MU/min for 10 MV FFF for TB) might be a bottleneck for treatment efficiency, as the machine is operating at its highest achievable dose rate at approximately 96% of the beam‐on time. This is also reflected in the low max_GS and max_MLC, indicating that gantry and MLC speeds are reduced to maintain the required high dose rates during delivery. The longer beam‐on time observed for HC SBRT plans is consistent with prior studies evaluating lung SBRT cases^18^ and is likely driven by HC's lower maximum dose rate compared with TB, which is impactful for higher dose per fraction plans.

In contrast, for non‐SBRT treatments, HC demonstrated significantly lower beam‐on time and higher MF values relative to TB. This could result from its higher max_GS and max_MLC values combined with higher nominal maximum gantry and MLC leaf speeds; the lower dose rate demands of non‐SBRT delivery allow HC to achieve faster delivery through greater utilization of these parameters. Notably, HC achieved more than 50 s reduction in beam‐on time per fraction for non‐SBRT treatments, substantially reducing the duration patients spend alone on the treatment couch. This could greatly reduce patient anxiety (e.g. claustrophobia associated with immobilization mask^19^) and reduce the likelihood of anxiety‐related delays or treatment interruptions. Conversely, TB demonstrated an average reduction of more than 40 s in beam‐on time per fraction for SBRT treatments, which may help mitigate treatment inaccuracies associated with intra‐fraction motion. With the appropriate machine assignment, up to ∼40 minutes can be saved per machine for a busy treatment day of 45 plans, which could translate to increased capacity of up to 3 additional patients per machine per day.

The higher modulation observed in HC is consistent with findings from a multi‐site longitudinal study of more than 1000 plans by Jegal et al.^20^ This increased modulation flexibility may in addition be attributed to HC's dual‐layer MLC design, which support greater modulation flexibility. This trend was also evident within the SBRT/SRS subgroup, aligning with results reported for single‐fraction lung SBRT,^18^ hypo‐fractionated breast irradiation,^6^ and stereotactic treatments for brain metastases.^9^

Disease site‐specific analyses further support the relationship between treatment technique and the machine parameter that ultimately limits delivery efficiency. Disease sites with a predominance of conventional fractionation treatments, such as head and neck, generally exhibited greater utilization of gantry and MLC speeds, suggesting that delivery was more frequently constrained by modulation requirements. In contrast, disease sites with a higher proportion of SBRT treatments, particularly prostate, were characterized by substantially higher dose rate utilization and lower gantry and MLC speed utilization, indicating a shift toward dose rate limited delivery. The prostate cohort provides a particularly illustrative example of this relationship. When SBRT cases were excluded, differences in gantry and MLC speed utilization between HC and TB were no longer significant, suggesting that the previously observed disparities were driven primarily by the large SBRT population rather than inherent disease‐site characteristics. Correspondingly, differences in beam‐on time also disappeared, highlighting the strong connection between machine constraint utilization and overall treatment efficiency. Interestingly, abdomen and pelvis treatments demonstrated relatively high utilization of dose rate, gantry speed, and MLC speed simultaneously. One possible explanation is the heterogeneous composition of these disease sites, which included a mixture of SBRT and non‐SBRT treatments. As a result, these cohorts may represent intermediate delivery regimes in which both modulation requirements and dose‐rate limitations contribute meaningfully to overall treatment efficiency.

Machine‐specific operational considerations limited treatments of certain disease sites (e.g. spine, lung, and breast) on both machines. Due to longer delivery times for SBRT on HC and requirements for kV planar capabilities for triggered imaging, all breath‐hold SBRT at our institution are treated on TB systems to minimize patient fatigue and motion uncertainty. In addition, spine SBRT and brain SRS are delivered exclusively on Edge systems due to integration with ExacTrac Dynamic (Brainlab AG, Munich, Germany) for real‐time motion monitoring. Furthermore, treatments requiring non‐coplanar beams, larger fields, or six DoF image matching can only be delivered on TB systems, limiting the versatility of HC for certain cases. With the introduction of a 6 DoF couch in HC 5.0, some of the prior limitations in setup flexibility may be mitigated, potentially enabling a redistribution of site and procedure‐specific cases across platforms.

There are several additional considerations when comparing treatment delivery efficiencies between HC and TB systems. First, as a retrospective analysis, this study includes a limited number of patients who received treatments on both systems, reducing the opportunities for direct patient‐matched comparisons. Further prospective studies could include a subset of patients planned and treated on both systems to reduce confounding factors inherent to retrospective clinical data, such as re‐planning due to anatomical changes or patient setup difficulties on HC versus TB. Second, incorporating imaging time into the analysis could give a more comprehensive evaluation of patient's total time spent alone on table and further inform triaging strategies. Although possessing higher imaging speeds, HC (with Hypersight) may have increased reconstruction time due to the non‐simultaneous operations of reconstruction and acquisition, as well as its more computationally intensive iterative reconstruction algorithms. HC may also require more CBCT acquisitions given the absence of kV planar imaging. Other factors, including differences in matching tolerances (eg. SBRT vs. non‐SBRT), challenges in bony vs. soft tissue matching, and day‐to‐day anatomical variation, may further influence the manual components of the imaging time. In addition, analysis of pre‐imaging setup time (e.g. patient entry, positioning, and initial alignment) could provide insight into portions of treatment time not directly related to machine performance and help inform disease site and patient‐specific strategies to reduce overall treatment duration. Additional considerations include the generalizability of institution‐specific machine performance metrics, as differences in machine configurations, patient populations, planning practices, and clinical workflows may influence treatment delivery efficiency. Consequently, the absolute values reported in this study should be interpreted as a large scale real‐world institutional experience rather than universally applicable benchmarks. Nevertheless, the trajectory log analysis methodology presented here is broadly transferable, as it relies on routinely archived machine log files that are readily available on modern linear accelerators. Other institutions can apply the same framework to characterize machine utilization patterns within their own clinical environments and identify opportunities for workflow optimization and machine assignment.

## 5 CONCLUSIONS

This study presents a transferable trajectory log‐based methodology for evaluating treatment delivery efficiency by quantifying utilization of dose rate, gantry speed, and MLC speed in conjunction with beam‐on time and MF. Analysis of more than 4,300 VMAT plans demonstrated that treatment delivery efficiency is governed by different machine constraints depending on treatment technique, with non‐SBRT plans tending to be modulation‐constrained and SBRT/SRS plans more frequently dose rate‐constrained. These differences resulted in shorter beam‐on times for HC in most conventionally fractionated treatments, whereas TB demonstrated advantages for SBRT/SRS deliveries. Beyond comparing two treatment platforms, the proposed methodology provides a practical framework for characterizing machine utilization and delivery constraints using routinely available trajectory log files. Such information may help guide treatment machine assignment, optimize clinical workflow, and support future evaluations of emerging radiotherapy delivery systems.

## AUTHOR CONTRIBUTIONS

*Conception and design*: Ruiming Edmondson, Douglas Moseley, and James Kavanaugh. *Data collection*: Ruiming Edmondson, Douglas Moseley, and Satomi Shiraishi. *Data analysis*: Ruiming Edmondson and Douglas Moseley. *Manuscript drafting*: Ruiming Edmondson. *Edits and final approval of manuscript*: Ruiming Edmondson, Douglas Moseley, Satomi Shiraishi, and James Kavanaugh.

## CONFLICT OF INTEREST STATEMENT

The authors declare no conflicts of interest.

## Supporting information



Supporting Information

Supporting Information

## Data Availability

Research data are stored in an institutional repository and will be shared upon request to the corresponding author.
