# Supplementary material for: Log file‐based treatment efficiency analysis of TrueBeam and Halcyon delivery systems
Source: J Appl Clin Med Phys. 2026 Sep 14;27(9):e70802. doi: 10.1002/acm2.70802 (PMC13575492; doi:10.1002/acm2.70802)
Supplement: Supplementary file 1 [file ACM2-27-e70802-s002.docx]

**Supplemental Materials.** a) Beam-on time, b) beam-on time normalized by dose per fraction, and c) modulation factor for Halcyon (HC) and TrueBeam (TB), categorized by disease site.
